# Non-surgical Management of Locally Advanced Basal Cell Carcinoma of the Upper Extremity With Vismodegib

**DOI:** 10.7759/cureus.28479

**Published:** 2022-08-27

**Authors:** Austin R Swisher, Mark J Landau, Allison B Davila, Armando A Davila, Caryn Zagaynov, Christopher A Bobbitt, Darren S Leong, Alexander Y Chang, Walter T Chang

**Affiliations:** 1 Department of Surgery, University of California Riverside School of Medicine, Riverside, USA; 2 Department of Plastic Surgery, Loma Linda University Medical Center, Loma Linda, USA; 3 Department of Plastic Surgery, Kaiser Permanente Fontana Medical Center, Fontana, USA

**Keywords:** upper extremity, neoadjuvant, sonic hedgehog, basal cell carcinoma, vismodegib

## Abstract

Basal cell carcinoma (BCC) is a common skin malignancy that can present reconstructive challenges in patients with locally advanced diseases of the extremities. This article highlights three cases of locally advanced BCC of the extremities managed with vismodegib (Erivedge, Genentech). Vismodegib is a sonic hedgehog pathway (Shh) inhibitor approved by the FDA for use in metastatic or recurrent BCC. All three patients in our case series demonstrated significant clinical responses with reductions in tumor size which obviated the need for complex reconstructive surgery or amputation.

## Introduction

There are over two million new cases of basal cell carcinoma (BCC) in the United States annually, making it the most prevalent skin malignancy [1]. The treatment of choice for BCC in cosmetically sensitive areas (face, neck, hands, feet, and genital region) is Mohs micrographic surgery. Other surgical treatments include wide local excision or excision with circumferential, peripheral, and deep margin assessment [2]. Unfortunately, while most cases of BCC are easily treated with simple surgical excisions, there are cases of delayed presentation, with approximately 1,000 deaths annually attributed to the disease [1]. In these more advanced cases, other non-surgical treatment modalities including radiation, topical 5-fluorouracil, imiquimod, photodynamic therapy, intralesional methotrexate, or combined laser cryoimmunotherapy have been utilized, but with mixed results [3-6].

The sonic hedgehog (Shh) pathway is an important molecular regulator of patterning and growth in the embryonic period. Aberrations in the Shh pathway have been implicated in the carcinogenesis of multiple malignancies, including BCC, medulloblastoma, and rhabdomyosarcoma [7]. Specifically, overactivation of the Shh signaling pathway by either the inhibition of the transmembrane protein patched homolog 1 (PTCH) or the activation of the transmembrane protein smoothened (SMO) is thought to be a necessary molecular event in the pathogenesis of >90% of BCC [8, 9]. Vismodegib is an orally available agent targeting the Shh signaling pathway. It acts via competitive inhibition of SMO, resulting in the inactivation of GLI/2 transcription factors and decreased expression of downstream oncogenes. Currently, vismodegib is approved for treating metastatic BCC, recurrent disease after surgical failure, or disease not amenable to surgical or radiation treatment [10]. Vismodegib is reported to be overall well tolerated. Common side effects include muscle spasms, dysgeusia, fatigue, alopecia, and nausea. The vast majority of these are mild (grades 1-2). High grade adverse events (grade 3-4) are uncommon [11]. It is also a potent teratogen [12].

Delayed presentation of a large BCC involving an extremity is a relatively uncommon but therapeutically challenging clinical scenario in the fields of reconstructive hand, plastic, or orthopedic surgery. Surgical management may necessitate amputation or complex reconstructive procedures, often resulting in significant loss of function. Even with the utilization of Mohs micrographic surgery, margins can be wide and unpredictable. Data regarding the use of Shh pathway inhibitors to treat large, locally advanced BCC of the upper extremity remains limited. Here, we report three cases from our institution where the use of vismodegib in a neoadjuvant setting in patients with large BCC lesions on the upper extremities resulted in dramatic regression of disease, thereby allowing management with minor or no surgical intervention and maximal preservation of limb function. Using the most recent European consensus-based interdisciplinary guidelines, each case was defined as difficult to treat with locally advanced or metastatic BCC [13]. This study is unique in that all participants were elderly, had significant medical comorbidities, and presented with large biopsy-confirmed BCC of the upper extremity.

## Case presentation

This study included three patients referred to a Plastic and Hand Surgery clinic for locally advanced BCC of the upper extremity over a one-year period. No patients were recruited as part of this study.

Case 1

**Figure 1:**
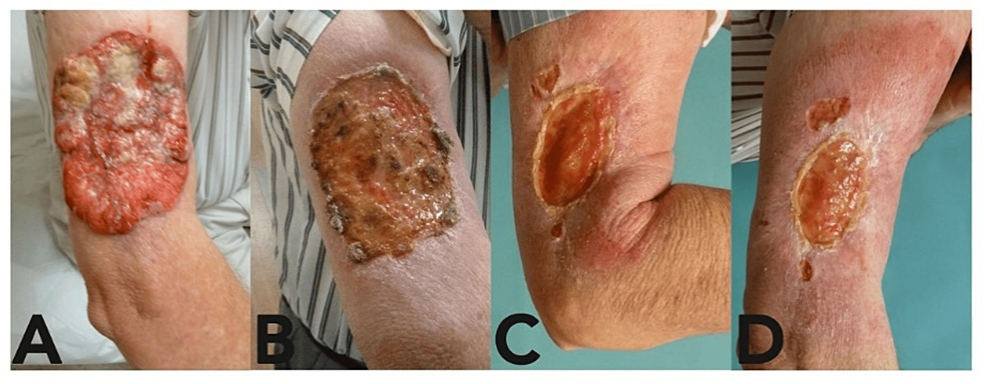
Progression of Case 1 with vismodegib treatment. A) Basal cell carcinoma at presentation; B) Lesion after three months of vismodegib; C) Lesion after seven months of vismodegib; D) Lesion after nine months of vismodegib.

A 67-year-old male with a history of diabetes mellitus and hypertension presented with a biopsy-proven 9 centimeter (cm) by 12 cm right lateral upper arm ulcerated BCC (nodular and infiltrative subtype), which had been neglected for years (Figure 1). CT imaging demonstrated infiltration of the tumor into the underlying musculature. This tumor was deemed unresectable without significant reconstructive surgery, which the patient declined. He was referred to Radiation Oncology and received radiation therapy (XRT) for three months. A follow-up examination three months after his initial presentation showed no change in the lesion size. The patient continued to decline surgery and was therefore started on vismodegib. After three months of vismodegib therapy, the lesion had decreased in size to 7.5 cm by 8 cm. The patient completed a total of nine months of vismodegib. His only reported side effect was dysgeusia, which resolved spontaneously after treatment completion. Thirteen months after his initial referral, re-examination revealed significant disease regression, with the lesion measuring 2 cm wide by 6 cm long. At the 16-month follow-up, mapping biopsies within and around the wound were obtained to determine resection margins prior to planned excision and grafting. All samples were pathologically negative for residual BCC. As such, the wound was treated conservatively with wound care and ultimately healed without evidence of recurrence.

Case 2

**Figure 2:**
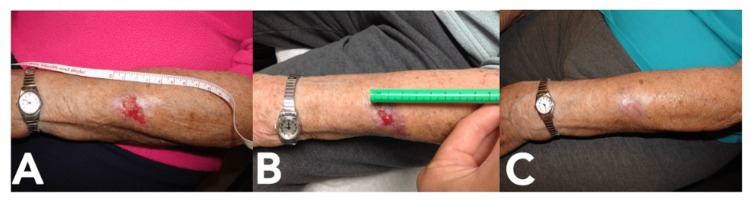
Progression of Case 2 with vismodegib treatment. A) Basal cell carcinoma at presentation; B) Lesion after one month of vismodegib; C) Lesion after three months of vismodegib.

A 90-year-old female with advanced Alzheimer’s dementia and multiple comorbidities presented with a 4 cm by 2.5 cm biopsy-proven BCC of the left forearm (Figure 2). She was referred to Plastic Surgery due to her complex medical history and the potential need for reconstruction with grafting. The patient was unable to cooperate with awake surgical treatment due to her mental status. She was also deemed a poor candidate for general anesthesia. It was unlikely that she would be able to perform the subsequent post-operative care required for any grafting or closure. Therefore, in three months, she started on vismodegib and had complete resolution of her lesion. This was confirmed by biopsy. She did not report any side effects from her therapy.

Case 3

**Figure 3:**
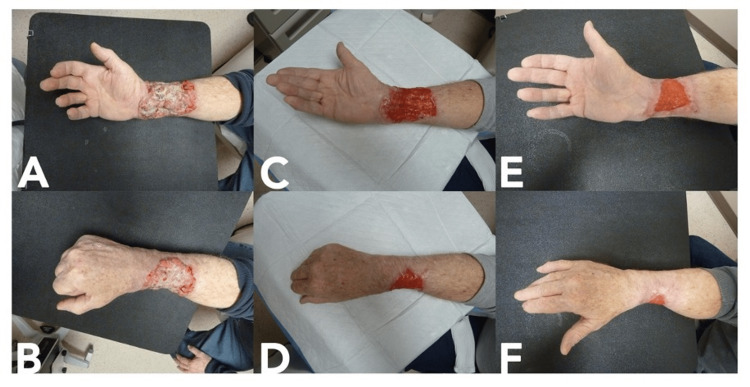
Progression of Case 3 with vismodegib treatment. A, B) Basal cell carcinoma at presentation; C, D) Lesion after one month of vismodegib; E, F) Lesion after three months of vismodegib

**Figure 4:**
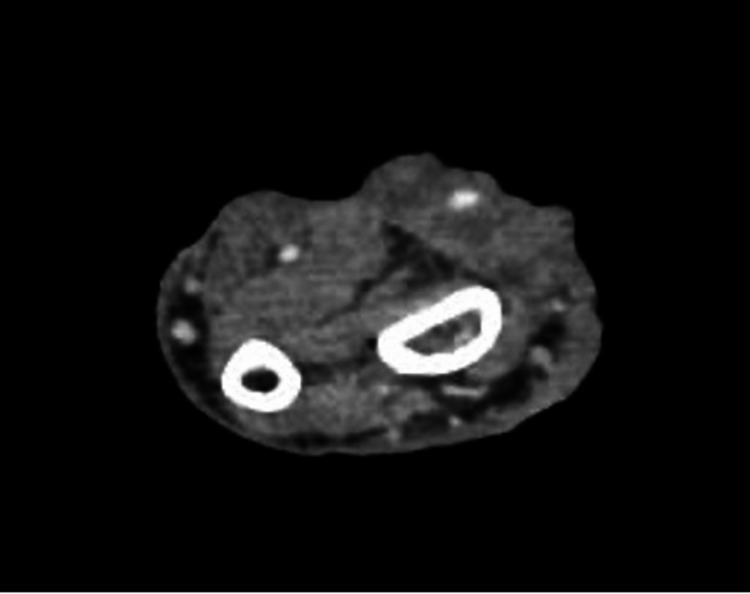
CT imaging of Case 3 prior to vismodegib treatment, demonstrating encasement of the radial artery.

A 65-year-old male with a history of cerebrovascular disease presented with multiple soft tissue masses, including a 4.5 cm by 2.5 cm upper back dark nodule and a near circumferential mass on the right distal third of the forearm measuring 15 cm by 12 cm (Figure 3). Biopsies of the arm lesion revealed BCC (nodular and infiltrative subtype), and a CT scan of the right upper extremity showed encasement of the radial artery (Figure 4). Excisional biopsy of the back lesion revealed melanoma, and a positron emission tomography (PET)/CT was consistent with metastasis to the ipsilateral axillary lymph nodes. The patient refused further surgical management of his melanoma and would not accept amputation of the right arm for management of the BCC. He was therefore started on vismodegib. After three months of treatment, his BCC was significantly reduced in size (from 15x12 cm initially to 8x7 cm at the last follow-up). He reported muscle spasms during therapy which was managed with amlodipine. He was scheduled for excision and reconstruction with a flow-through free flap; however, he passed away from his metastatic melanoma prior to surgery.

## Discussion

Vismodegib (Erivedge) is an orally available medication that received FDA approval in 2012 for treating BCC that is locally invasive, metastatic, or recurrent [12]. In follow-up studies, the clinical benefit of vismodegib in patients with advanced BCC was reported in 76% of patients [14]. It is also approved for use in lesions where resection or radiation would cause substantial disfigurement or loss of function and in patients who are not candidates for surgery or XRT. Median relapse-free survival was reported as 18.4 months; most patients who experienced relapse still responded to a vismodegib rechallenge [15]. Switching hedgehog inhibitors may also benefit patients with resistant BCC [16].

Since its introduction, vismodegib has demonstrated effectiveness in treating BCC of diverse origins, including cosmetically sensitive and surgically challenging regions, such as the nose and periorbita [17, 18]. Other studies have reported the benefits of using vismodegib to reduce tumor mass prior to surgical excision or radiotherapy [19, 20]. For example, in a study of 55 patients with locally advanced BCC, 80% achieved surgical downstaging after neoadjuvant vismodegib, with downstaging defined by the severity of aesthetic and functional consequences of surgery [21]. Case reports have also demonstrated the effectiveness of vismodegib in patients with BCC of the face secondary to xeroderma pigmentosum, a condition that often precludes surgery as an option [22, 23].

Chemoresistance secondary to mutations in the Shh pathway, specifically in the transmembrane SMO, can limit vismodegib’s effectiveness [8]. Since statistically significant tumor size reduction has been demonstrated in as short as three months, Ching JA et al. suggested monitoring patients for their response to hedgehog inhibitor therapy every three months to decide whether surgery may be indicated during therapy [24]. Reflectance confocal microscopy has been shown to be a sensitive technique for identifying subclinical residual BCC during and after the completion of vismodegib treatment [25]. Multidisciplinary teams, including dermatology, oncology, radiation oncology, and surgery, could effectively coordinate care and determine whether patients with BCC would be best treated medically, surgically, or as a combination of both.

Many oncologic and reconstructive surgeons, however, are unfamiliar with the potential of vismodegib to improve resectability and reduce the need for complex reconstructions. This may be particularly beneficial in patients with delayed presentation of neglected BCCs involving the extremities. These patients often have complex medical histories or social challenges that make them poor candidates for complex surgical interventions which require high levels of patient participation and compliance. Our results suggest that vismodegib can be safely tolerated in elderly patients with multiple comorbidities.

## Conclusions

Tumors involving the upper extremity present a unique challenge given the relative lack of local tissue for reconstruction and the functional importance of the hand. There remains a dearth of information regarding the indications and outcomes of vismodegib use in hand tumors, and long-term follow-up data regarding efficacy and adverse effects are lacking. More research should be conducted to compare survival rates and patient-centered outcomes of emerging medical and surgical modalities for treating BCC of the hand and forearm. In our series, we were able to achieve reductions in surgical morbidity and improved preservation of function in all three patients with the pre-operative use of this medical therapy. We hope our experience will add to the increasing recognition of this useful medical adjunct in the Hand Surgery community.
